# Nanotechnological Research for Regenerative Medicine: The Role of Hyaluronic Acid

**DOI:** 10.3390/ijms25073975

**Published:** 2024-04-03

**Authors:** Flavia Carton, Manuela Malatesta

**Affiliations:** Department of Neurosciences, Biomedicine and Movement Sciences, University of Verona, Strada Le Grazie 8, 37134 Verona, Italy; flavia.carton@univr.it

**Keywords:** hyaluronic acid, hyaluronan, tissue engineering, hydrogel, nanoparticle, cartilage regeneration, bone regeneration, wound healing, angiogenesis, neural regeneration

## Abstract

Hyaluronic acid (HA) is a linear, anionic, non-sulfated glycosaminoglycan occurring in almost all body tissues and fluids of vertebrates including humans. It is a main component of the extracellular matrix and, thanks to its high water-holding capacity, plays a major role in tissue hydration and osmotic pressure maintenance, but it is also involved in cell proliferation, differentiation and migration, inflammation, immunomodulation, and angiogenesis. Based on multiple physiological effects on tissue repair and reconstruction processes, HA has found extensive application in regenerative medicine. In recent years, nanotechnological research has been applied to HA in order to improve its regenerative potential, developing nanomedical formulations containing HA as the main component of multifunctional hydrogels systems, or as core component or coating/functionalizing element of nanoconstructs. This review offers an overview of the various uses of HA in regenerative medicine aimed at designing innovative nanostructured devices to be applied in various fields such as orthopedics, dermatology, and neurology.

## 1. Introduction

**Figure 1:**
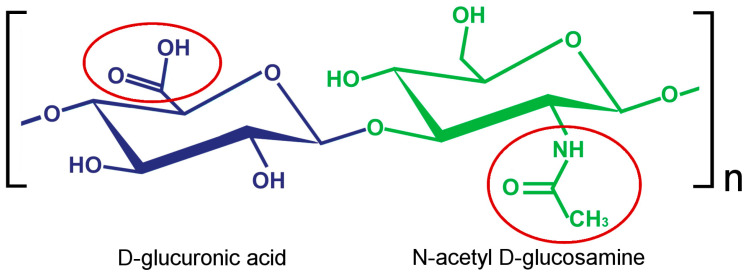
Chemical structure of hyaluronic acid; red circles indicate groups for binding with different chemical compounds.

Hyaluronic acid (HA), also known as hyaluronan or hyaluronate, is a linear, anionic, non-sulfated glycosaminoglycan made of thousands of disaccharides (N-acetyl D-glucosamine and D-glucuronic acid) linked via β-glycosidic bonds (Figure 1). This polysaccharide occurs in almost all body tissues and fluids of vertebrates including humans. It is a main component of the extracellular matrix (ECM) and, thanks to its high water-holding capacity, plays a major role in water transport, tissue hydration, and maintenance of osmotic pressure [1]. Due to its hygroscopic and viscoelastic properties, HA acts as a lubricant in body fluids, such as the vitreous [2] and synovial ones [3]. HA is also involved in cell proliferation, differentiation, adhesion and migration [4,5,6,7,8], inflammation [9], immunomodulation [10,11,12], antioxidant response [13,14,15], tissue regeneration [16,17,18], and angiogenesis [19,20,21]. Remarkably, the cytoprotective potential of HA is related to the polymer length; the higher the molecular mass, the stronger the protection from different stressors. In particular, it has been demonstrated that high-molecular-mass HA exerts antioxidant effects not only by scavenging reactive oxygen species (ROS) in the ECM but also by triggering intracellular cytoprotective response via CD44 signaling [22].

Based on these properties and its natural origin, which make it non-toxic, non-allergic, non-sensitizing, and physiologically degradable via oxidative species and hyaluronidases, HA has found extensive application in medicine and cosmetics (e.g., [23,24,25,26,27,28,29,30]). In particular, regenerative medicine, which aims at “replacing or regenerating human cells, tissue or organs, to restore or establish normal function” [31], has been a fruitful field of research on HA and its derivatives because of their multiple physiological effects on tissue repair and reconstruction. Tissue engineering led to the production of a large variety of novel HA-based biomaterials mostly aimed at mimicking the microenvironment of ECM. In this view, various manufacturing techniques were set up to obtain physiologically relevant hydrogels [32] characterized by tunable physicochemical properties (e.g., stiffness, strength, viscosity, shear thinning, self-healing), responsiveness to specific external or internal stimuli, and capability of controlled release of biological factors, thus optimizing the growth, adhesion, migration, and proliferation of the embedded cells. In addition, these advanced manufacturing techniques allowed the production of HA hydrogels with improved biocompatibility and degradability, thus favoring their successful implantation in vivo [33,34]. These HA-based devices were applied in various medical fields giving successful results: in orthopedics, by promoting tissue regeneration and reducing inflammation in osteochondral lesions, osteoarthritis, and tendinopathies [35,36,37]; in dentistry, by improving wound healing after oral and maxillofacial surgery as well as in periodontics, endodontics, and dental implants [38,39]; in dermatology, by treating skin damage and injury [40,41,42]; in neurology, by helping nervous system healing in neurological disorders or after stroke [43,44,45]; in cardiovascular medicine, by recovering injured myocardial tissue [46]; in ophthalmology, by stimulating cornea repair [47].

**Figure 2:**
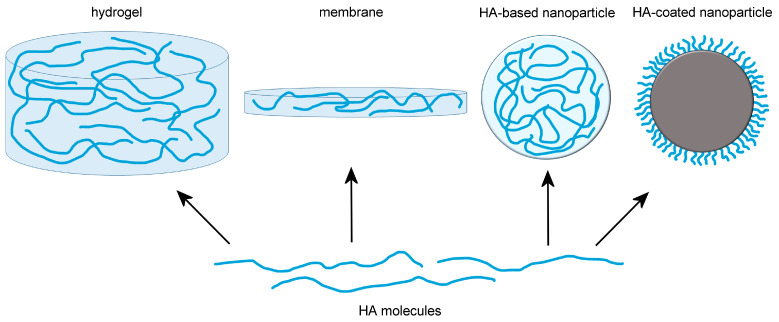
In nanoformulations for regenerative medicine, hyaluronic acid (HA) has been used as the main component of multifunctional hydrogels and membranes, or as a core or coating element of nanoparticles.

In recent years, nanotechnology has been integrated in tissue engineering: organic and inorganic nanomaterials (nanoparticles, nanotubes, nanocrystals, etc.) have been used either as nanofillers to improve the architecture and compactness of hydrogels allowing modulation of the swelling and degradation rate, or as functional elements (e.g., nanocarriers for growth factors, cytokines, drugs; magnetic or electroconductive nanoconstructs) to ameliorate the proliferation, differentiation, and migration of the encapsulated cells [34]. Consequently, nanomedical formulations containing HA as the main component of multifunctional hydrogels systems, or as a core component or coating/functionalizing element of nanoconstructs have been set up for regenerative purposes (Figure 2).

The present review is intended to offer a general overview of innovative HA-based nanostructured devices designed to promote natural healing. By reporting the nanotechnological strategies set up to improve the regenerative properties of HA, the review outlines the current state of the art of this research field with a special focus on the biomedical results, and casts a glance at the prospects for the use of nanostructured HA in regenerative medicine.

## 2. Nanomedical Applications of HA for Cartilage Regeneration

Cartilage regeneration after injury is still a great challenge in clinics because of some characteristics of this tissue such as avascularity, low cellularity, and poor migration ability of chondrocytes.

An interesting article reports the capability of HA-based hydrogels to enhance articular cartilage repair due to the ability of HA to regulate inflammation, improve the lubricity of cartilage boundaries, promote the adhesion, proliferation, and differentiation of chondrocytes, and support limb bud formation via cartilage cell-matrix deposition [48].

In recent years, several strategies have been developed to functionalize HA-based hydrogels in order to enhance the chondrogenesis of encapsulated stem cells and chondrocytes, regulate the inflammatory microenvironment, and promote ECM regeneration of cartilage [48]. For example, Riveiro et al. [49] developed an HA hydrogel crosslinked with divinyl sulfone and reinforced with lithium bioactive glass micro- and nanofibers produced by laser spinning. This innovative hydrogel, characterized by enhanced elastic modulus and suitable to deliver therapeutic lithium-ions, was proved to promote the proliferation and the chondrogenic behavior of the mouse ATDC5 cell line in vitro. Galarraga et al. [50] developed different norbornene-modified HA hydrogels reinforced with polycaprolactone microfibers produced by melt-electrowriting to investigate the influence of hydrogel’s crosslink density on nutrient transport, new matrix distribution, and stability. In vitro and ex vivo results proved that the loosely crosslinked hydrogels were able to better support the chondrogenesis of encapsulated mesenchymal stem cells (MSCs), promote the deposition and distribution of ECM, and enhance neocartilage formation compared to more densely crosslinked hydrogels.

In other studies, composite HA-based hydrogels have been developed as platforms to deliver nanoparticles (NPs) and bioactive drugs. Chen et al. [51] prepared a biocompatible cellulose nanocrystal/gelatin-methacrylate anhydride/HA-methacrylateanhydride-blended hydrogel, integrated with synthetic melanin-based NPs and the bioactive drug kartogenin, with the aim to provide a sustained release of this agent. In vitro and in vivo assessments demonstrated the successful release of kartogenin in enhancing the proliferation of extracellular bone-derived MSCs and, remarkably, their differentiation into chondrocytes. Hong et al. [52] set up a composite scaffold made of poly-L-lysine/kartogenin (L-K) NPs and poly(lactic-co-glycolic acid) (PLGA)/methacrylated HA (PLHA) able to facilitate chondrogenic differentiation and cartilage regeneration; they developed this novel platform by the self-assembling of polymer, thus avoiding complex chemical processes, and improved a new fabrication process of PLHA via coprecipitation and UV crosslinking. In vitro tests proved the biocompatibility and the efficient internalization of NPs into adipose-derived stem cells. L-K NPs also increased the expression of chondrogenesis-related genes and, after 12 weeks, newly formed cartilage resembling native cartilage was observed in vivo in rabbits [52]. Similarly, Yan et al. [53] developed an injectable UV-polymerizable HA hydrogel with kartogenin-loaded PLGA NPs able to facilitate hyaline cartilage and subchondral tissue repair in a porcine model at 12-month follow-up. Kim et al. [54] developed an HA hydrogel able to guarantee a sustained release of tauroursodeoxycholic acid (TUDCA) from PLGA microspheres fabricated using a water-in-oil-in-water double emulsion solvent evaporation technique. In more detail, they developed a bilayer plug loaded with two different concentrations of TUDCA-PLGA microspheres for the controlled release of TUDCA to favor the regeneration of osteochondral defects. Biological assays highlighted the simultaneous regeneration of cartilage and bone tissue indicating that the TUDCA bilayer plug may be a promising candidate scaffold for generating a subchondral-mimicking tissue composed of the cartilage and bone tissue simultaneously [54].

Platelet-rich plasma (PRP) has great regenerative properties by reason of hundreds of bioactive molecules (growth factors and cytokines) released by activated platelets [55]. Notably, these factors are released as free molecules or enclosed in microvesicles, which act as micro/nanostructured vectors [56,57]. The therapeutic potential of PRP in facilitating cartilage healing has been exploited by Yan et al. [58], who combined autologous PRP with photo-crosslinkable injectable HA hydrogel integrated with PLGA NPs loaded with kartogenin. In vivo studies performed in minipigs showed that, at 6 months after operation, this treatment led to a better development of hyaline-like cartilage in terms of ECM and type II collagen, without the formation of hypertrophic cartilage, indicating that the combined used of HA and PRP ameliorates cartilage healing likely due to the sustained release of platelet factors to the surrounding tissues.

In another study [59], MSCs were encapsulated inside injectable thermosensitive poly(N-isopropylacrylamide)/HA hydrogels containing various amounts of chitosan-gacrylic acid-coated PLGA micro/nanoparticles prepared by a single emulsion solvent evaporation technique. This hydrogel showed a great integration with the natural cartilage and improved growth and proliferation of the encapsulated MSCs. Moreover, histological analysis of glycosaminoglycan (GAG) showed that melatonin, a chondrogenic factor, was able to improve GAG synthesis making this hydrogel a promising candidate for cartilage tissue engineering.

The bioactive properties of HA may be improved by combining HA with native ECM microparticles (i.e., demineralized bone matrix and decellularized cartilage) and hydroxyapatite NPs. This viscoelastic colloidal gel was therefore made exclusively of natural components. An in vitro study of this matrix on human umbilical cord MSCs demonstrated its biocompatibility and ability to support cell viability making it a promising tissue filler potentially suitable for articular cartilage repair [60].

Finally, a composite hydrogel made of gelatin methacrylate/HA methacrylate was developed for annulus fibrosus injuries [61]. In detail, mesoporous silica NPs decorated with ceria and transforming growth factor β3 (TGF-β3) were added to the composite hydrogel to provide antioxidant and anti-inflammatory properties, and enhance the recruitment of annulus fibrosus cells. Results obtained in annulus fibrosus cultured cells and in a rat model proved that this hydrogel was able to regulate the microenvironment by clearing ROS, inhibiting inflammation, and inducing macrophage M2 polarization. Moreover, the release of TGF-β3 improved annulus fibrosus cells recruitment and ECM deposition, thus improving in situ tissue repair [61].

**Figure 3:**
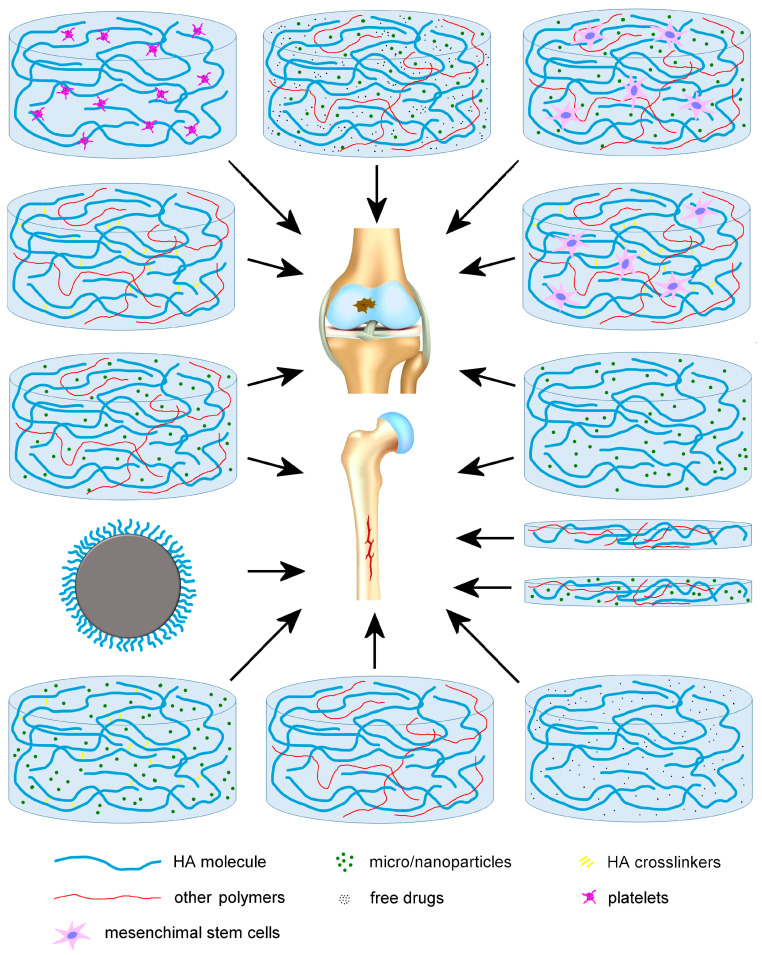
Schematic illustration of the different hyaluronic acid (HA)-based nanoformulations developed for cartilage and bone regeneration.

The nanoformulations described in this section are illustrated in Figure 3.

## 3. Nanomedical Applications of HA for Bone Regeneration

Bone tissue engineering is a rapidly developing field driven by the need to face bone disorders and injuries such as osteoporosis, bone defects, and fractures caused by trauma or disease. In this context, HA-based hydrogels have gained a great amount of attention because of their ability to provide a suitable microenvironment for bone regeneration. As a matter of fact, HA can interact with growth factors involved in bone formation such as bone morphogenic proteins and TGF-β. HA can also interact with specific cell receptors, such as CD44 and the receptor for HA-mediated motility, CD168, expressed on bone forming and shaping cells such as osteoblasts, osteoclasts, and MSCs [62].

In recent research on innovative tools to improve bone regeneration, HA has been used alone or combined with other biomaterials such as collagen, chitosan, silk fibroin, gelatin, and synthetic polymers [63]. Arjama et al. [64] developed an injectable hydrogel based on functionalized composite of HA and hydroxyapatite, crosslinked with silk fibroin. This novel approach increased the mechanical strength of the hydrogel making it similar to native bone. The agents present in the hydrogel were able to improve the osteogenic activities required for hard tissue development such as osteoblast attachment, communication, and maturation. Similarly, Noh et al. [65] developed an injectable hydrogel as a 3D-printable bioink using three biocompatible biomaterials: HA, hydroxyethyl acrylate, and gelatin methacryloyl. This hydrogel was obtained firstly by graft polymerization of HA and hydroxyethyl acrylate, and then by grafting of gelatin methacryloyl via radical polymerization mechanism. A series of in vitro evaluations proved that this hydrogel had good biocompatibility and viability for MC3T3 bone cells. In addition, the incorporation of bioactive agents (e.g., growth factors, chemical agents, or genetic materials) into HA-based hydrogels showed great promise in enhancing the hydrogel’s properties for bone regeneration. Rajabnejadkeleshteri et al. [66] developed a composite scaffold made of gelatin, elastin, and HA containing pectin microparticles microfluidicly loaded with bone morphogenetic protein-2 (BMP-2)-conjugated carbon dots. This hydrogel system achieved sustained release of BMP-2 and enhanced the pro-osteogenic effect in vitro on the human osteosarcoma cell lines, MG-63.

Successful results have repeatedly been obtained by combining an HA-based scaffold with other biomaterials or bioactive agents improving bone regeneration in both in vitro and in vivo models. For example, Flegeau et al. [67] developed a silanized HA and hydroxypropylmethylcellulose hydrogel incorporating biphasic calcium phosphate granules to improve their mechanical properties as osteoconductive biomaterials. In vivo assays performed in a rabbit knee defect model proved that HA/biphasic calcium phosphate composites were fully degraded and beneficially influenced bone regeneration by increasing the space available for bone ingrowth and by accelerating the biphasic turnover of calcium phosphate granules. Kisiel et al. [68] proposed a human BMP-2 (rhBMP-2) delivery system based on an integrin-specific ligand (fibronectin fragment) covalently grafted to HA hydrogel. This hydrogel improved stem cell attachment and spreading, and enhanced the osteogenic potential of rhBNP-2.

Composite HA-based hydrogels have been also developed as NP delivery systems for osteoregeneration. Zhang et al. [69] fabricated a crosslinked aldehyde HA/N,O-carboxymethylchitosan-based hydrogel doped with a sphingosine 1-phosphate loaded inside alginate/chitosan polyelectrolyte-modified silica NPs prepared via electrostatic interaction. This formulation improved the mechanical properties of the hydrogel and allowed sustained release of the angiogenic drug, thus providing a favorable support for bone marrow MSCs adhesion and proliferation, and stimulating the recruitment of endothelial cells via microvascular formation. A novel core/shell, bio-inspired, drug-loaded polymeric 3D-printed scaffold made of gelatin, polyvinyl alcohol and HA integrated with doxycycline/hydroxyapatite/polycaprolactone NPs was successfully developed for osteoregeneration [70]. This new biopolymer-based hydrogel with improved mechanical properties was able to support healing of bone tissue by sustainedly releasing doxycycline and enhancing proliferation, mineralization, and osteodifferentiation of MSCs. Hong et al. [71] developed a self-crosslinked thiolated HA injectable hydrogel integrated with biphasic calcium phosphate NPs prepared by disulfide crosslinking. Cell viability and proliferation assays performed in MC3T3-E1 osteoblast cells revealed the biocompatibility and the osteogenic potential of this hydrogel making it a promising scaffold for bone tissue engineering. Jamnezhad et al. [72] developed an alginate-HA bone filler using freeze drying technique for orthopedic applications. This nanocomposite was reinforced with titanium dioxide NPs that induced antibacterial properties decreasing the risk of infection, and accelerated the healing process. The hydrogel was nontoxic for osteoblast cells except for the highest incorporation of titanium dioxide NPs. Zhou et al. [73] exploited the immunomodulatory potential of quercetin by formulating a new composite hydrogel using quercetin-loaded solid lipid NPs homogeneously packed into an HA hydrogel combined with a temperature-responsive poly(e-caprolactone-co-lactide)-b-poly(ethylene glycol)-b-poly(e-caprolactone-co-lactide) polymer. This scaffold was able to create an immune microenvironment responsible for promoting the recruitment and differentiation of bone marrow MSCs, stimulating angiogenesis and regulating osteoclast homeostasis. Dennis et al. [60] developed a viscoelastic colloidal gel for potential use as bone filler defects by combining native ECM microparticles with HA and hydroxyapatite NPs: in vitro assays demonstrated the biocompatibility of the gel on human umbilical cord MSCs, making it a promising platform to repair a wide variety of bone defects.

HA has also been used as a film on the surface of NPs to support bone tissue regeneration. Amorim et al. [74] reported the construction of a poly-L-lysine and HA film onto charged surfaces of silica NPs using the layer-by-layer technique. The biological activity of these bilayer coating was then evaluated on human bone marrow stem cells demonstrating that, at appropriate concentrations, these NPs were able to improve the in vitro osteogenic differentiation without affecting cell viability and proliferation.

Finally, adhesive and osteoconductive membranes made of HA have been developed for hard tissue regeneration. Fonseca et al. [75], inspired by marine mussels’ adhesive proteins, developed an HA/chitosan membrane modified with catechol groups using the compaction of polyelectrolyte complexes method, in order to enhance the adhesive properties of the assembled membranes. Moreover, ternary bioactive glass NPs (BGNPs) have been entrapped into the membrane to stimulate mineralization. Mechanical and in vitro studies revealed that the composition of this membrane possessed bioactive capabilities, enhancing mechanical and adhesive properties and increasing activity of SaOs-2 osteoblast-like cells, thus making this system suitable to improve bone regeneration [75]. In a similar way, Almeida et al. [76] developed a multilayered film made of BGNPs as the inorganic phase, and HA and chitosan catechol conjugates as the organic phase. In vitro biological tests performed on L929 mouse fibroblast cells showed that these multilayered films had a positive influence on the cellular response contributing to ameliorate cell viability, proliferation, and adhesion, and suggested that this system may be proficiently used for orthopedic implants [76].

The nanoformulations described in this section are illustrated in Figure 3.

## 4. Nanomedical Applications of HA for Wound Healing

HA-based hydrogels represent ideal candidates for improving wound healing. The unique biological properties of HA are involved in each sequential phase of the wound healing process such as hemostasis, inflammation, proliferation, and maturation. In this context, HA exhibits important functions including skin moisturization, tissue repair, hemostatic properties, regulation of inflammation and resistance to bacterial adhesion. Thus, as a consequence of these attractive advantages, HA-based hydrogels have been successfully developed as multifunctional scaffold for a wide clinical application in wound repair [77].

Nanotechnological research contributed to expanding the potential of HA as a regenerative tool for wound healing through the development of HA-based hydrogels as localized drug-delivery systems.

Tan et al. [78] developed a dual drug-loaded nanocomposite polysaccharide-based self-healing hydrogel formed by the dynamic imine bonds and electrostatic interactions between carboxymethyl chitosan and oxidized HA. This polymer matrix was enriched by curcumin-loaded mesoporous polydopamine NPs and metformin to promote diabetic wound healing. A combined study including in vitro assays on blood, L929 fibroblast cells and *Staphylococcus aureus* or *Escherichia coli* colonies, and in vivo tests in a murine model proved that this hydrogel was able to exert an effective antibacterial activity and support re-epithelization, the formation of the granulation tissue, angiogenesis, collagen deposition and arrangement, as well as wound contraction in diabetic wound healing [78].

Similarly, using a reactive mixing bioprinting approach, Puertas-Bartolomé et al. [79] prepared a hydrogel bioink to produce 3D scaffolds made of carboxymethyl chitosan and HA, and containing catechol functionalized polymeric NPs loaded with coumarin-6. In vitro studies demonstrated that the bioprinted scaffolds supported viability and proliferation of encapsulated L929 fibroblasts over 14 days, indicating that this bioink may be a promising material for application in skin tissue regeneration processes [79].

Mousavi Nejad et al. [80] developed two types of hybrid systems. In detail, they synthetized a hybrid system based on either double crosslinked HA and PLGA/dexamethasone sodium phosphate NPs or on HA hydrogel/dexamethasone sodium phosphate without NPs. Results proved that the hydrogel containing NPs showed a controlled dexamethasone release improving the proliferation rate of HFFF2 human fibroblast cells. Overall, the data obtained suggested that this hybrid system could represent a good candidate for intraperitoneal drug delivery systems, anti-adhesion barriers, and wound healing applications.

The potential of small extracellular vesicles (sEV) to promote wound healing and inhibit scar tissue hyperplasia has been exploited, loading sEV inside HA-based hydrogels [81]. In particular, the hydrogel was prepared by crosslinking carbohydrazide-modified gelatin with oxidized HA through the Schiff base reaction under mild conditions; then, polydopamine NPs and MSC-secreted sEV were added to obtain a dual-nanoagent-loaded multifunctional hydrogel. In vitro assays performed on human keratinocyte (HaCaT), human umbilical vein endothelial (HUVEC) and mouse fibroblast (L929) cell lines, and in vivo studies in a murine model demonstrated that the proposed scaffold was able to efficiently release sEV thus enhancing cell viability, proliferation, migration, and colony formation, and modifying the cell cycle and apoptosis rate. Additionally, this hydrogel was able to promote skin re-epithelization after radiation damage, thus attenuating the pathological microenvironment in vivo.

To control and prolong the release of drugs, HA was used as an NP coating, thus enhancing a stable and long-lasting repair effect on wound healing. Lin et al. [82] described a new type of pH-sensitive wound dressing obtained by combining a hybrid gelatin methacrylate system with chlorhexidine-loaded silica NPs coated with HA. In vitro analysis demonstrated that this hydrogel provided an ultra-long-acting drug release with no cytotoxic effect on L929 fibroblast cells and a sustained antibacterial activity against *S. aureus* and *E. coli*. Moreover, in vivo experiments in a murine model demonstrated the capability of this system to promote skin wound healing, opening interesting perspectives for its therapeutic use.

**Figure 4:**
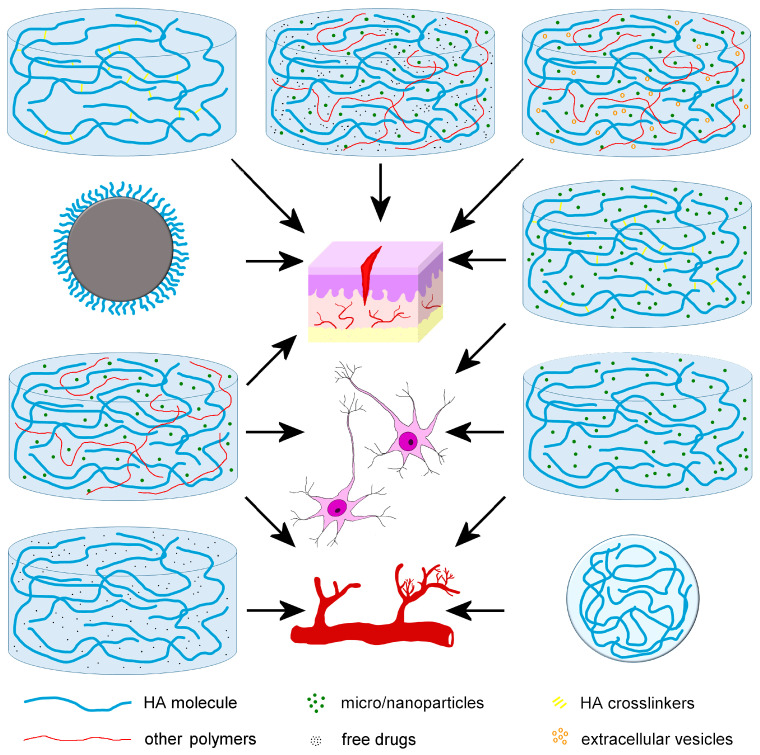
Schematic illustration of the different hyaluronic acid (HA)-based nanoformulations developed to promote wound healing, angiogenesis, and nervous tissue regeneration.

The nanoformulations described in this section are illustrated in Figure 4.

## 5. Nanomedical Applications of HA for Angiogenesis

HA-based hydrogels developed for delivering angiogenic factors proved to have a significant potential in several clinical applications, such as wound healing, myocardial infarction, limb ischemia, brain injuries, and bone repair. HA promotes angiogenesis by enhancing endothelial cell proliferation, migration, and tube formation; in addition, HA influences metalloproteinase activity and contributes to the stability of new formed microvessels by prompting the deposition of ECM components (e.g., collagen) by fibroblasts [83]. On this basis, HA hydrogels have been used to develop novel nanotools to improve neo-vascularization in regenerative medicine applications.

A novel platform system composed of tyramine-modified HA hydrogel incorporating star-shaped or linear poly(glutamic acid)-based NPs loaded with stromal-derived Factor 1α (SDF-1α) was recently developed with the aim of making this powerful angiogenic protein suitable for clinical applications [84]. In fact, a system able to release SDF-1α in a localized and sustained manner would overcome the problem of the short half-life of this factor in vivo. Results demonstrated that, thanks to the ability of HA hydrogel to act as a delivery vehicle, the released SDF-1α could interact with the CXC chemokine receptor of HUVEC cells, thus activating the phosphatidylinositol 3-kinase/protein kinase B signaling pathway required to increase the formation of tubules by the endothelial cells and the tubules’ length. A similar nano-in-gel delivery system was developed by the same research group to deliver the vascular endothelial growth factor (VEGF) through polyglutamic acid NPs [85]. Results demonstrated that this hydrogel was able to improve blood vessel number and branching in an in vivo chick chorioallantoic membrane model. Moreover, in vitro assays performed on HUVEC cells proved the ability of the system to enhance cell migration and improve the total tubule length.

Another nanohybrid HA-based hydrogel was developed by Jian et al. [86] to deliver SDF-1α and basic fibroblast factor (bFGF) loaded into sulfated glycosaminoglycan-based polyelectrolyte complex NPs. The purpose was to enhance the recruitment of endogenous neural stem cells and regulate their fate. The potential of SDF-1α and bFGF to enhance both angiogenesis and neurogenesis was demonstrated both in cultured rat neuronal stem cells (HCN-A94-2) and in in vivo ischemic stroke models induced by photothrombosis in cortical blood vessels.

Vignesh et al. [87] designed an injectable chitosan-HA-based hydrogel to deliver the pro-angiogenic molecule, deferoxamine. In detail, this drug was loaded into PLGA NPs prepared by a double emulsion solvent diffusion technique; then the NPs were entrapped inside the chitosan-HA hydrogel. In vitro studies showed excellent biocompatibility and an increased proliferation rate in rat adipose-derived stem cells as well as in HUVEC cells. Moreover, the hydrogels system containing deferoxamine-loaded NPs improved tube formation in HUVEC cells. These data have been also confirmed by experiments performed by subcutaneously injecting mice with the developed hydrogel. Results showed a maximal blood vessel formation by a stabilizing hypoxia inducible factor. These findings suggested that this hydrogel may be used to stimulate angiogenesis in regenerative medicine.

In the context of endodontic regenerative therapies, Silva et al. [88] developed injectable HA-based hydrogels formed in situ, reinforced with cellulose nanocrystals and enriched with platelet lysate. This scaffold was used to sustainably deliver chemotactic and pro-angiogenic growth factors i.e., the platelet-derived growth factor (PDGF) and VEGF, with the aim to enhance the revascularization of the root canal system and regenerate the dentin-pulp complex. The platelet lysate-loaded hydrogel stimulated the chemotactic and pro-angiogenic activity by promoting cell recruitment and sprouting in co-cultures of human dental pulp cells and HUVEC cells, and in an ex vivo model performed through the chicken chorioallantoic membrane assay.

HA has also been used in the form of NPs to stabilize and enhance the regenerative performance of bio-scaffolds. For instance, Mondalek et al. [89] used HA-poly(de-co-glycolide) NPs to improve the physical and biological properties of porcine-derived small intestine submucosa that was used as a bio-scaffold. In vitro and in vivo tests demonstrated that this biomaterial was able to enhance endothelial cell proliferation and promote angiogenesis in a preclinical large animal bladder augmentation model.

The nanoformulations described in this section are illustrated in Figure 4.

## 6. Nanomedical Applications of HA for Nervous Tissue Regeneration

HA represents a useful material for nervous tissue engineering. In particular, in the central nervous system HA has been shown to play an important role due to its ability to reduce the inflammatory response at the site of injury, improve neuronal growth, and inhibit glial scar formation [90,91].

Serafin et al. [90] developed an HA-based hydrogel as a delivery platform to promote regeneration in cases of spinal cord injury. In detail, NPs made of conductive polymers, such as poly(3,4-ethylenedioxythiophene) (PEDOT), were synthesized via chemical oxidation polymerization in miniemulsion, and then incorporated within a scaffold made of gelatin and HA. The presence of these NPs increased the conductivity and improved the mechanical properties of the hydrogel system, which proved to be biocompatible for MSCs and a rat spinal cord injury model. In vitro assessment showed an upregulation of the glial fibrillary acidic protein (GFAP), a sensitive marker related to the inflammatory process, around the periphery of the lesion/implantation site and a diminished astrocyte reactivity. These data proved the ability of the scaffold to regulate the initiation of the regeneration processes via decreased levels of macrophages and microglia at the implant site. Overall, the immunomodulatory potential of HA enabled the hydrogel to positively influence the microenvironment for regeneration. Similarly, Wang et al. [92] produced PEDOT-based NPs doped with HA via chemical oxidant polymerization. These NPs were then introduced into a chitosan/gelatin matrix via chemical crosslinking to create a conductive matrix. This scaffold improved neural stem cell proliferation with the upregulated expression of Ki-67, and enhanced differentiation in neurons and astrocytes with upregulated expression of ß tubulin-III and GFAP, respectively.

Li et al. [91] developed a hydrogel made of HA hydrazide chains and laminin-derived PPFLMLLKGSTR peptide-modified aldehyde-HA chains; during gelation, MnO_2_ NPs were dispersed into this matrix forming a MnO_2_ NP-dotted HA hydrogel. In vitro and in vivo studies performed on MSCs derived from the amniotic membrane of human placenta and in long-span rat spinal cord transfection models proved the ability of this scaffold to increase stem cell adhesion and growth as well as nerve tissue bridging, and to alleviate the oxidative environment, thus improving MSCs viability.

Führmann et al. [93] developed an injectable hydrogel made of crosslinked furan-modified HA as a delivery platform for the bioactive brain-derived neurotrophic factor (BDNF) loaded into PLGA NPs. Notably, this hydrogel was obtained by click-crosslinking, which provide stable gels avoiding cytotoxic agents and side products. Results showed that this hydrogel was safe and biocompatible in an experimental rat model of spinal cord injury, and allowed a sustained release of BDNF in vitro, significantly improving neurite outgrowth in rat embryo dorsal root ganglia: this opens promising perspectives for this platform as a therapeutic tool for spinal cord injuries. Similarly, Jian et al. [86] developed a nanohybrid hydrogel from HA matrices to control the delivery of SDF-1α and bFGF to modulate the cellular responses after nervous system injury. Briefly, the two binding proteins were incorporated into polyelectrolyte complex NPs, and their release was controlled in response to matrix metalloproteinase. It was observed that, after hydrogel injection, the migration, accumulation, and proliferation of endogenous neural stem cells was enhanced, thus improving the regeneration of the infarcted brain tissues. Moreover, the therapeutic efficacy of the matrix was evaluated in a rat model of ischemia stroke, showing that the local delivery of SDF-1 α and bFGF not only improved endogenous neurogenesis but also promoted angiogenesis in the peri-infarction tissue.

The nanoformulations described in this section are illustrated in Figure 4.

## 7. Conclusions

The successful application of HA in regenerative medicine has been known for a long time thanks to the multiple physiological roles that HA plays in cell growth and differentiation, and in inflammation.

As shown by the present review, the advent of nanotechnology in tissue engineering has determined the development of novel HA-based systems with considerably improved regenerative potential; the majority of these systems have been designed for applications in orthopedics, dermatology, and neurology, and mostly consisted of HA-based multifunctional scaffolds containing nanoconstructs as structural/functional constituents, whereas HA-based nanoparticles were rarely developed.

This is probably just the beginning of a new era in the exploitation of the potential of HA in regenerative nanomedicine. Some meta-analyses and systematic reviews have already highlighted the good performance of some next-generation HA-based devices in different regenerative applications (e.g., [94,95,96]). The new nanotechnology-based manufacturing processes are greatly expanding the potential of these next-generation HA hydrogels. Stimuli-responsive HA-materials may be programmed to precisely direct therapeutic outcomes in specific disease/injury conditions or in specific patient groups. Moreover, tunable and stimuli-responding HA hydrogels may provide a solution for complex regeneration processes characterized by spatiotemporal signaling. These achievements could pave the way for the application of HA-based nanodevices in precision medicine, personalized medicine, and even theranostics in many medical fields, including regenerative medicine.

Despite prolific research activity, the complex and costly fabrication of new biomaterials prevents the transfer to large scale production, making clinical translation difficult and creating an important bench-to-bedside gap. Moreover, regulatory bodies need a long time to approve the use of biomaterials for human or animal medicine. Finally, biomaterials need to be simple in design but they are intended to recreate native tissues and must be integrated into the extreme structural and functional complexity of the living organism; this represents a great challenge for researchers and the results achieved so far are often suboptimal.

Despite—or thanks to—these limitations, in the coming years tissue engineering will surely see an increase in nanotechnological reinterpretations of HA in a growing number of medical fields, with the consequent development of innovative regenerative treatments based on this multifaceted molecule.

## Data Availability

The data presented in this study are available upon request from the corresponding author.
